# Leveraging Ecological Momentary Assessment Data to Characterize Individual Mobility: Exploratory Pilot Study in Rural Uganda

**DOI:** 10.2196/54207

**Published:** 2024-06-10

**Authors:** Aleya Khalifa, Laura K Beres, Aggrey Anok, Ismail Mbabali, Charles Katabalwa, Jeremiah Mulamba, Alvin G Thomas, Eva Bugos, Gertrude Nakigozi, Larry W Chang, M Kate Grabowski

**Affiliations:** 1 Department of Epidemiology Mailman School of Public Health Columbia University New York, NY United States; 2 ICAP at Columbia University New York, NY United States; 3 Department of International Health Bloomberg School of Public Health Johns Hopkins University Baltimore, MD United States; 4 Rakai Health Sciences Program Kalisizo Uganda; 5 Department of Epidemiology University of North Carolina Chapel Hill, NC United States; 6 Department of Surgery Johns Hopkins University Baltimore, MD United States; 7 Pritzker School of Medicine University of Chicago Chicago, IL United States; 8 Department of Population Family and Reproductive Health Johns Hopkins Bloomberg School of Public Health Baltimore, MD United States; 9 Department of Internal Medicine University of Pittsburgh Medical Center Pittsburgh, PA United States; 10 Department of Epidemiology Bloomberg School of Public Health Johns Hopkins University Baltimore, MD United States; 11 Division of Infectious Diseases Department of Medicine Johns Hopkins School of Medicine Baltimore, MD United States; 12 Department of Pathology Johns Hopkins School of Medicine Baltimore, MD United States

**Keywords:** ecological momentary assessment, spatial analysis, geographic mobility, global positioning system, health behaviors, Uganda, mobility, pilot study, smartphone, alcohol, cigarette, smoking, promoting, promotion, alcohol use, cigarette smoking, mobile phone

## Abstract

**Background:**

The geographical environments within which individuals conduct their daily activities may influence health behaviors, yet little is known about individual-level geographic mobility and specific, linked behaviors in rural low- and middle-income settings.

**Objective:**

Nested in a 3-month ecological momentary assessment intervention pilot trial, this study aims to leverage mobile health app user GPS data to examine activity space through individual spatial mobility and locations of reported health behaviors in relation to their homes.

**Methods:**

Pilot trial participants were recruited from the Rakai Community Cohort Study—an ongoing population-based cohort study in rural south-central Uganda. Participants used a smartphone app that logged their GPS coordinates every 1-2 hours for approximately 90 days. They also reported specific health behaviors (alcohol use, cigarette smoking, and having condomless sex with a non–long-term partner) via the app that were both location and time stamped. In this substudy, we characterized participant mobility using 3 measures: average distance (kilometers) traveled per week, number of unique locations visited (deduplicated points within 25 m of one another), and the percentage of GPS points recorded away from home. The latter measure was calculated using home buffer regions of 100 m, 400 m, and 800 m. We also evaluated the number of unique locations visited for each specific health behavior, and whether those locations were within or outside the home buffer regions. Sociodemographic information, mobility measures, and locations of health behaviors were summarized across the sample using descriptive statistics.

**Results:**

Of the 46 participants with complete GPS data, 24 (52%) participants were men, 30 (65%) participants were younger than 35 years, and 33 (72%) participants were in the top 2 socioeconomic status quartiles. On median, participants traveled 303 (IQR 152-585) km per week. Over the study period, participants on median recorded 1292 (IQR 963-2137) GPS points—76% (IQR 58%-86%) of which were outside their 400-m home buffer regions. Of the participants reporting drinking alcohol, cigarette smoking, and engaging in condomless sex, respectively, 19 (83%), 8 (89%), and 12 (86%) reported that behavior at least once outside their 400-m home neighborhood and across a median of 3.0 (IQR 1.5-5.5), 3.0 (IQR 1.0-3.0), and 3.5 (IQR 1.0-7.0) unique locations, respectively.

**Conclusions:**

Among residents in rural Uganda, an ecological momentary assessment app successfully captured high mobility and health-related behaviors across multiple locations. Our findings suggest that future mobile health interventions in similar settings can benefit from integrating spatial data collection using the GPS technology in mobile phones. Leveraging such individual-level GPS data can inform place-based strategies within these interventions for promoting healthy behavior change.

## Introduction

The emerging field of human mobility highlights 2 key considerations for health research: individuals’ “neighborhoods” extend beyond where they live to other parts of their activity space and spatial determinants of health may vary in both their prevalence and their effect across these activity spaces [1]. Despite growing knowledge in high-income countries [2,3], there remains a dearth of data in low- and middle-income countries (LMICs) relating individual-level mobility to individual-level health behaviors.

Understanding people’s activity spaces (the places visited and the activities that take place there) can inform health promotion design and implementation. For example, one of the few GPS-based human mobility studies in the region used individual-level data to inform malaria programs in Zimbabwe as to whether bed nets should be delivered to households or high-mobility areas during the rainy season [4]. The mostly rural district of Rakai, Uganda, is characterized by substantial mobility for fishing and trading, among other family and school-related reasons [5-7], demonstrating significant time spent away from home. While evidence suggests that fisherfolk may be more likely to drink alcohol if they reside nearer to community drinking venues [8], without information on activity spaces beyond households, it is unclear whether similar behavior patterns exist in spaces away from home such as where people socialize or conduct commerce. GPS data collected from consenting individuals using mobile health (mHealth) apps, like ecological momentary assessments (EMA), offer an opportunity to analyze individual activity spaces vis-à-vis health behaviors like drinking alcohol.

This study analyzed individual mobility and spatial locations of health behaviors—all reported on an EMA app—to explore relationships between health and place and demonstrate the potential utility of such data for informing future place-based interventions.

## Methods

### Overview

A pilot trial using EMA messaging to influence certain health-related behaviors was conducted in Rakai, Uganda in 2017 [9]. In addition to supporting healthful behaviors (eg, fruit and vegetable consumption), an EMA app sent real-time messages to support users in reducing smoking, drinking alcoholic beverages, and having condomless sex with a non-long-term partner. Participants aged 18-49 years with a telephone number and who completed at least secondary education were purposively recruited from the Rakai Community Cohort Study (RCCS), an ongoing population-based cohort, between February 2016 and March 2017 [5]. Participants were given a password-protected smartphone with the trial’s app (emocha Health Inc) installed, along with a charger, power bank, and funds to purchase 525 megabytes of data monthly [9]. As part of the informed consent process, participants were told that the smartphone would track their location over the study period and that the same phone would be used to log their health behaviors—all of which would be kept confidential in locked files or secure computers by authorized project personnel.

This substudy used 2 data types: GPS locations and user-initiated reports. The smartphone app logged GPS coordinates every 1-2 hours if the phone was turned on and connected to the internet, otherwise, data were logged once the phone was connected. Using the app, participants were instructed to submit self-initiated behavioral reports within 1 hour of engaging in any of the behaviors. Each participant’s user-initiated reports were linked to logged GPS coordinates by nearest timestamp. Sociodemographic information from the most recently available RCCS survey (2015-2018) was used to describe the study population [10]. Participants’ home GPS coordinates were logged during RCCS household enumeration.

For each participant, the great circle distance was measured in kilometers between each recorded GPS point and the previously occurring point [11]. GPS points were excluded if they were outside of sub-Saharan Africa (<1% of GPS coordinates per participant). Interpoint distances were totaled over each participant’s roughly 90-day observation period [12]. Mobility was summarized as the average distance traveled per week to account for certain days of the week with outliers of distance traveled (eg, staying home on Sundays) that can bias centrality estimates [13]. Two other measures were also described: the number of unique places visited (deduplicated points within 25 m of one another, based on smartphones’ 5-20-m GPS point accuracy [12]), and the percent of GPS points recorded away from home [7]. To calculate the latter measure, GPS points were dichotomized as being located within or outside a home buffer region. A 100-m Euclidian distance circular buffer around participants’ home GPS coordinates reflected the home location, accounting for physical landscape and GPS accuracy [14]. We also calculated 400-m and 800-m buffers reflecting home neighborhoods that might include neighbors’ residences, shops, and transit stops [15].

We further evaluated the number of unique locations where behaviors occurred and the number of behavior locations away from the home or home neighborhood among participants reporting a particular behavior, using the same methods as above [16].

### Ethical Considerations

The Ugandan Virus Research Institute Research and Ethics Committee and the Johns Hopkins School of Medicine institutional review board approved the pilot trial, registered on ClinicalTrials.gov (NCT04375423). Written informed consent was obtained from interested participants at the first study visit. Any identifying information (eg, contact information) was stored in locked files or on secured computers. Once study data were collected, each participant was assigned a unique identifier and all data for analyses were deidentified. Participants were compensated for their time with 10,000 UGX (approximately US $3 at the time) and refunded for their travel to each study visit [9].

## Results

A total of 46 EMA trial participants had both RCCS and GPS data, and 34 of 80,131 total GPS points from across 9 participants were excluded because they were outside of sub-Saharan Africa, potentially indicating international travel or device malfunction. Participants contributed GPS points across a median of 65 (IQR 45-78) observation days, at a median of 23 (IQR 18-31) points per day. Participants were mostly of higher socioeconomic status and lived in an agrarian or trading community (Table 1).

**Figure 1:**
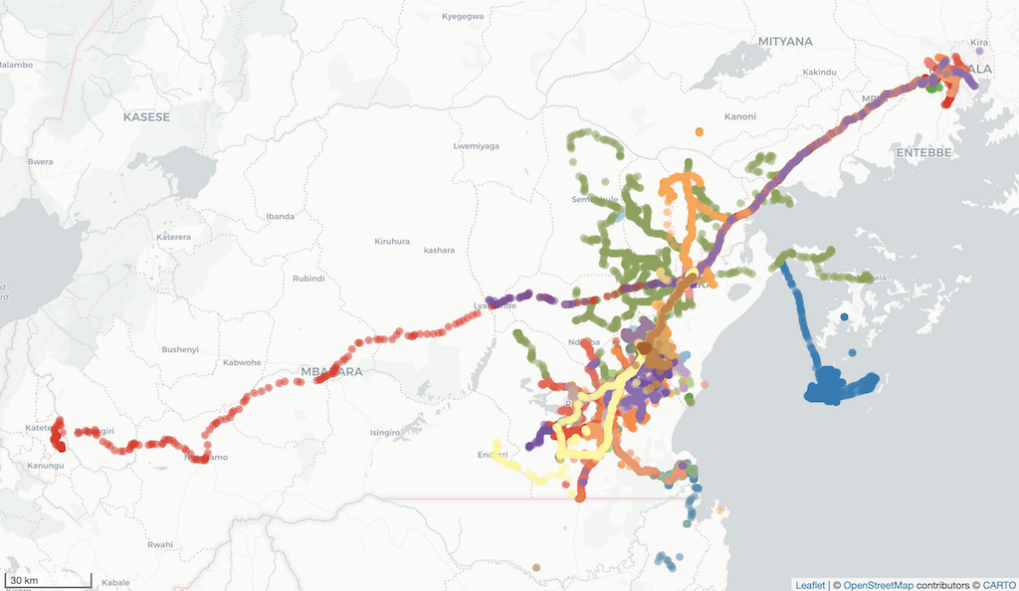
Activity paths among pilot trial participants in Rakai, Uganda, 2016-2017, (N=46). Each color represents a unique participant’s activity path. The figure was created using OpenStreetMap and CARTO. OpenStreetMap is licensed under the Open Data Commons Open Database License [17].

Participants’ activity spaces varied. Common paths included travel to or from Kampala (Uganda’s capital city) and Masaka City (the largest city in south-central Uganda), local travel around Rakai district, and travel along Lake Victoria coastlines and islands (Figure 1 [17]). The median weekly distance traveled was 303 (IQR 152-585 km; Table 2). While participants contributed on median of 1292 (963-2137) GPS points, they reported a median of 389 (IQR 218-706) unique locations over their 90-day observation period. A median of 92% (IQR 75%-97%), 76% (IQR 58%-86%), and 67% (IQR 51%-82%) of participants’ GPS points were outside of their 100-m, 400-m, and 800-m home buffer regions, respectively.

Of the participants reporting alcohol use, cigarette smoking, and condomless sex with a non–long-term partner, each behavior was reported a range of 1-72, 1-37, and 1-18 times, respectively (Table 2), and 17 (74%), 5 (55%), and 8 (57%) participants reported those behaviors at 2 or more unique locations (Table 3). Of the participants reporting, alcohol use, smoking, and condomless sex were reported 0-70, 1-36, and 1-11 times away from the home location (ie, outside the 100-m buffer), respectively. Participants reported all behaviors slightly less frequently outside the home neighborhood (ie, outside the 400-m and 800-m buffers).

**Table 1:** Sociodemographic characteristics from the Rakai Community Cohort Study, 2015-2018, among pilot trial participants in Rakai, Uganda (N=46).

Characteristic			Participants, n (%)
**Gender**			
	Men	24 (52)	
	Women	22 (48)	
**Age group (years)**			
	20-24	14 (30)	
	25-34	16 (35)	
	35-45	16 (35)	
**Socioeconomic status quartile**			
	Lowest	7 (15)	
	Low-middle	6 (13)	
	High-middle	18 (39)	
	Highest	15 (33)	
**Highest level of education**			
	Secondary	34 (74)	
	Tertiary	12 (26)	
**Reported migration since the last survey round**			
	No migration	37 (80)	
	In-migrated	2 (4.3)	
	Out-migrated	7 (15)	
**Type of community**			
	Agrarian	21 (46)	
	Fishing	5 (11)	
	Trading	20 (43)	
**Primary occupation**			
	Agriculture	15 (36)	
	Retail/trading/vending	7 (17)	
	Construction/transportation	2 (4.8)	
	Government/clerical/teaching	12 (29)	
	Student	1 (2.4)	
	Other	5 (12)	
	Unknown	4	
**Vehicle access**			
	Access to car or motorcycle	18 (39)	
	Access to a bicycle	20 (43)	

**Table 2:** Measures of mobility and health behavior locations among pilot trial participants in Rakai, Uganda, 2016-2017 (N=46).

Measures			Median (IQR)		Range
Distance traveled (km) per week			303 (152-585)		15-6962
GPS points (n)			1292 (963-2137)		68-7157
Unique locations visited (n)			389 (218-706)		62-1752
GPS points outside 100-m home buffer region (%)			92 (75-97)		<1-100
GPS points outside 400-m home buffer region (%)			76 (58-86)		<1-100
GPS points outside 800-m home buffer region (%)			67 (51-82)		<1-100
**Alcohol use (n=23, 50% of individuals reported)**					
	Reports, n	4.0 (2.5-8.5)		1-72	
	Unique locations where behavior occurred (n)	3.0 (1.5-5.5)		1-23	
	Reports away from home (outside 100-m buffer region; n)	3.0 (1.5-5.0)		0-70	
	Reports away from home (outside 400-m buffer region; n)	2.0 (1.0-4.0)		0-43	
	Reports away from home (outside 800-m buffer region; n)	2.0 (0.0-3.0)		0-27	
**Cigarette smoking (n=9, 20% of individuals reported)**					
	Reports, n	4.0 (1.0-6.0)		1-37	
	Unique locations where behavior occurred (n)	3.0 (1.0-3.0)		1-20	
	Reports away from home (outside 100-m buffer region; n)	2.0 (1.0-5.0)		1-36	
	Reports away from home (outside 400-m buffer region; n)	2.0 (1.0-3.0)		0-22	
	Reports away from home (outside 800-m buffer region; n)	1.0 (1.0-3.0)		0-5	
**Condomless sex with a non–long-term partner (n=14, 30% of individuals reported)**					
	Reports, n	6.0 (1.0-9.5)		1-18	
	Unique locations where behavior occurred (n)	3.5 (1.0-7.0)		1-16	
	Reports away from home (outside 100-m buffer region; n)	3.0 (1.0-9.0)		1-11	
	Reports away from home (outside 400-m buffer region; n)	2.5 (1.0-6.75)		0-10	
	Reports away from home (outside 800-m buffer region; n)	2.5 (0.25-6.0)		0-10	

**Table 3:** Summary statistics of health behavior locations among pilot trial participants in Rakai, Uganda, 2016-2017 (N=46).

Summary statistics		Participants, n (%)
**Alcohol use (n=23, 50% of individuals reported)**		
	One unique location	6 (26)
	Two or more unique locations	17 (74)
	Any report away from home (outside 100-m buffer region)	22 (96)
	Any report away from home (outside 400-m buffer region)	19 (83)
	Any report away from home (outside 800-m buffer region)	16 (70)
**Cigarette smoking (n=9, 20% of individuals reported)**		
	One unique location	4 (44)
	Two or more unique locations	5 (55)
	Any report away from home (outside 100-m buffer region)	9 (100)
	Any report away from home (outside 400-m buffer region)	8 (89)
	Any report away from home (outside 800-m buffer region)	8 (89)
**Condomless sex with a non–long-term partner (n=14, 30% of individuals reported)**		
	One unique location	6 (43)
	Two or more unique locations	8 (57)
	Any report away from home (outside 100-m buffer region)	14 (100)
	Any report away from home (outside 400-m buffer region)	12 (86)
	Any report away from home (outside 800-m buffer region)	10 (71)

## Discussion

### Principal Findings

EMA trial participants were highly mobile, with over 75% of participants traveling at least 150 km/week. Most participants reporting cigarette smoking, alcohol use, and condomless sex with a non–long-term partner recorded those behaviors in 2 or more unique locations. mHealth studies using mobile phone apps could leverage GPS-based mobility data from consenting participants to inform future interventions.

The high mobility observed in this sample reflects the regional context, known for transient fisherfolk communities, localized market trading, and transport corridors connecting Kampala to the Tanzanian border [5]. As expected, participants traveled between Rakai town and Masaka city (approximately 65 km), Kampala (approximately 197 km), and fish landing sites around Misozi (approximately 60 km). In comparison, residents of a rural agricultural district of Zimbabwe traveled a fraction of the distance per week [4]. Place-based interventions should consider the local socioeconomic context when deciding where and how to deliver services, as it seems to impact the scale and geographic patterning of individual mobility [18].

Since most participants reported health behaviors in 2 or more unique locations, EMA interventions could consider using strategies, like geofencing, to deliver behavior change messages when users enter geographies in which they have historically been more likely to report certain behaviors [19,20]. For example, individuals may prefer to drink alcohol in locations where consumption is acceptable, affordable, and available [8]. Geofencing is understudied in LMICs but has been used in South Africa, where CareConekta (a GPS-based mHealth app) links people living with HIV to health facilities while traveling outside their regular clinic catchment area [21].

Increased availability of and access to mHealth technologies with GPS capabilities, including increasing mobile connectivity in LMICs, can make spatial analyses possible [22]. Our study demonstrates successful data collection using study-supplied mobile phones with an EMA app [23]. This means spatial data collection and analysis can be more easily integrated into mHealth studies than others that provide a separate wearable GPS device for short periods [24].

### Limitations

This study has limitations. First, the sample may not accurately represent the underlying population as only individuals with secondary education or higher were eligible to ensure they could be trained on how to use the smartphone app. For example, these findings may overrepresent the mobilities and health behaviors of people in higher socioeconomic levels. Second, under half of the participants reported each behavior, and often only once, limiting the analysis. Third, great circle distance measures may underestimate travel distances [25], though short distances between GPS points (median 0.8 km) likely minimized this bias. Finally, Euclidian buffer regions may not accurately reflect neighborhoods. Road networks and geographic features, or participant interviews, could also be used to define areas that more accurately reflect what individuals perceive as their “neighborhood” [1].

### Conclusions

As a growing number of mHealth interventions in LMICs attempt to apply place-based strategies to improve health outcomes [26-30], more work is needed to understand the mobility of potential users. Among residents in rural Uganda, as part of a pilot trial, an EMA app successfully captured high mobility and health-related behaviors in multiple locations. Future mHealth interventions in similar settings could leverage individual-level GPS data to inform place-based strategies for promoting healthy behavior change.

## Abbreviations

EMA: ecological momentary assessment
LMIC: low- and middle-income country
mHealth: mobile health
RCCS: Rakai Community Cohort Study

## Data Availability

The data sets analyzed during this study are not publicly available due to the sensitive nature of individual-level GPS data and data sharing policies of the Rakai Health Sciences Program (RHSP) but are available from RHSP on reasonable request (datarequests@rhsp.org).
